# Prevalence and determinants of opioid use disorder among long-term opiate users in Golestan Cohort Study

**DOI:** 10.1186/s12888-023-05436-x

**Published:** 2023-12-21

**Authors:** Saba Alvand, Masoumeh Amin-Esmaeili, Hossein Poustchi, Gholamreza Roshandel, Yasaman Sadeghi, Vandad Sharifi, Farin Kamangar, Sanford M. Dawsey, Neal D. Freedman, Christian C. Abnet, Afarin Rahimi-Movaghar, Reza Malekzadeh, Arash Etemadi

**Affiliations:** 1 Liver and Pancreaticobilliary Research Center, Digestive Disease Research Institute, Tehran University of Medical Sciences, Tehran, Iran; 2 Department of Mental Health, Johns Hopkins Bloomberg School of Public Health, Baltimore, MD USA; 3 Iranian National Center for Addiction Studies (INCAS), Tehran University of Medical Sciences, Tehran, Iran; 4 Golestan Research Center of Gastroenterology and Hepatology, Golestan University of Medical Sciences, Gorgan, Iran; 5 Department of Psychiatry, Roozbeh Hospital, Tehran University of Medical Sciences, Tehran, Iran; 6 Department of Biology, School of Computer, Mathematical, and Natural Sciences, Morgan State University, Baltimore, MD USA; 7 Metabolic Epidemiology Branch, Division of Cancer Epidemiology and Genetics, National Cancer Institute, NIH, Bethesda, MD 20892 USA; 8 Digestive Oncology Research Center, Digestive Disease Research Institute, Tehran University of Medical Sciences, Tehran, Iran

**Keywords:** Cohort studies, Iran, Opiate, Opioid use disorder, Opium, Substance-related disorders

## Abstract

**Background:**

Number of opiate users worldwide has doubled over the past decade, but not all of them are diagnosed with opioid use disorder. We aimed to identify the prevalence and risk factors for OUD after ten years of follow-up.

**Methods:**

Among 8,500 chronic opiate users at Golestan Cohort Study baseline (2004–2008), we recalled a random sample of 451 subjects in 2017. We used three questionnaires: a questionnaire about current opiate use including type and route of use, the drug use disorder section of the Composite International Diagnostic Interview lifetime version, and the validated Kessler10 questionnaire. We defined opioid use disorder and its severity based on the DSM-5 criteria and used a cutoff of 12 on Kessler10 questionnaire to define psychological distress.

**Results:**

Mean age was 61.2 ± 6.6 years (84.7% males) and 58% were diagnosed with opioid use disorder. Starting opiate use at an early age and living in underprivileged conditions were risk factors of opioid use disorder. Individuals with opioid use disorder were twice likely to have psychological distress (OR = 2.25; 95%CI: 1.44–3.52) than the users without it. In multivariate regression, former and current opiate dose and oral use of opiates were independently associated with opioid use disorder. Each ten gram per week increase in opiate dose during the study period almost tripled the odds of opioid use disorder (OR = 3.18; 95%CI: 1.79–5.63).

**Conclusions:**

Chronic opiate use led to clinical opioid use disorder in more than half of the users, and this disorder was associated with psychological distress, increasing its physical and mental burden in high-risk groups.

**Supplementary Information:**

The online version contains supplementary material available at 10.1186/s12888-023-05436-x.

## Introduction

The United Nations Office on Drugs and Crime (UNODC) reported that the number of opiate users has doubled over the past decade [1]. In 2019, 61.6 million people used opioids worldwide, of whom 30.8 million used minimally-processed products (i.e. opiates such as heroin and opium), with the remainder using synthetic or semi-synthetic preparations [1]. Although all opiate users are at risk of adverse health conditions, not all of them fulfil the criteria for dependence or abuse, and they should be evaluated individually with clinical criteria for opioid use disorder (OUD) [2]. Between 1990 and 2016, the prevalence of OUD increased by 47·3%, making it the most common drug use disorder in the world [3]. History of opioid-related poisoning, the opioid dose used, polysubstance use, a diagnosis of psychiatric disorders, and the influences of the social environment are currently known risk factors for opioid use disorder [4–6]. However, diagnosing psychiatric disorders is not feasible in a large scale study of the general population [7, 8]. For these reasons, information on risk factors of OUD and patterns of long-term use in the general population is relatively scarce.

Consumption of minimally-processed opium is a known carcinogen [9] and is associated with an increased overall risk of cancer [10] cardiovascular diseases [11], and mortality [12]. Afghanistan produces more than 80% of the total illicit opium in the world [13], and UNODC has predicted a rise in illicit cultivation in Afghanistan due to the economic crisis following the COVID pandemic [1]. Iran is a transit route for trafficking opium and its derivatives from Afghanistan to the rest of the world and accounts for 42% of worldwide consumption of opium [14]. Opium consumption in Iran dates back thousands of years and opium abuse surged in this country in the late 15th century [15]. Long-term use of opium and its derivatives are still common in Iran [16], and it is sometimes regarded as a “soft drug” due to its widespread use [17]. The 2011 Iranian Mental Health Survey showed an OUD prevalence of 2.23% in the 15- to 64-year-old population [18]. This is while the global age-standardized rate of opioid dependence was 0.51% (510 per 100,000 people) in the year 2017 [19].

The current study used baseline and follow-up data from the Golestan Cohort Study (GCS), accrued from the population of Golestan Province in northeastern Iran. GCS started in January 2004, in the context of an international collaboration, initially intending to identify risk factors for upper gastrointestinal cancers due to the high incidence of esophageal cancer in this area, as detailed before [20]. Among more than 50,000 participants, 8487 (17%) reported long-term use of opium at baseline based on a validated structured questionnaire [12]. In 2017, we used the GCS infrastructure to recall a group of these individuals. This nested study is the first longitudinal study aiming to determine the prevalence and potential risk factors of OUD including demographics, habitual history, psychological distress, and opiate use characteristics in long-term opium users after ten years of follow-up.

## Materials and methods

### Participants

Informed consent was obtained from all participants. All procedures were performed in compliance with relevant laws and institutional guidelines and that the National Institutes of Health (NIH) IRB has approved this study (protocol number: 07CN120). The GCS originally recruited 50,045 individuals, aged 40–75, from the general population of Golestan Province in northeastern Iran between 2004 and 2008. In the GCS, individuals were randomly selected through systematic clustering based on household numbers and invited by telephone contact. Details of the GCS have been published before [20]. In 2017, about 13 years after GCS baseline enrolment, we administered a repeat comprehensive opiate and tobacco use questionnaire, along with validated psychological tools detailed below, to investigate the psychological correlates of long-term opiate use. We invited a random sample of 500 participants who reported chronic opium use at baseline, stratified by sex and tobacco use history. To select this random sample, we complied the list of all participants who were alive and reported opiate use at baseline and categorizing them into 4 strata defined by current tobacco use and sex. Since these two factors are important determinants of opiate use, from each stratum we selected a number of individuals proportional to their prevalence among all GCS participants, to ensure representativeness. Data from 451 subjects who accepted to participate were analyzed for the present study. This was more than the required sample size of 385 calculated based on a type 1 error of 0.05, a 50% prevalence of OUD among users and a 5% error margin.

### Measurements

The baseline information was derived from demographic and lifestyle data collected by trained interviewers in the GCS. The lifestyle questionnaire included questions about substance and tobacco use. The questionnaire included history of substance use over a person’s life, dates of starting and stopping, types of drugs used, routes, and self-reported doses of opiate use in grams. In this province, the main substance of abuse is opium which is used in two forms: teriak (raw opium) and shireh (a refined product extracted by boiling opium pipe residue in water, with or without adding teriak), which are taken by oral or smoking routes. The quantity measure for opium use has been previously shown to be valid against urinary codeine and morphine with 93% sensitivity and 89% specificity [21]. History of any lifetime alcohol use was also obtained from all participants. Alcohol consumption is relatively uncommon in this population (ever use less than 5%), and most ever users have had a limited number of drinks for a short period of time. There were no current alcohol drinkers in our study sample, and we defined the consumption of 1 drink or more per week at any period during a person’s life as a history of ever alcohol use.

We used three different questionnaires in this study: [1] a detailed questionnaire asking about current opiate type, dose and route, [2] to define OUD we used the validated L section of the Farsi translation Composite International Diagnostic Interview (CIDI 2.1-lifetime version) [22, 23], and [3] the validated Farsi version of the Kessler 10 (K10) questionnaire [7, 8] to define psychological distress. The questionnaires were administered by two trained interviewers after a workshop and a pilot phase supervised by two investigators who were involved in the development and validation of the Farsi version of the substance use and K10 questionnaires [24].

We defined lifetime OUD based on the Diagnostic and Statistical Manual of Mental Disorders, Fifth Edition (DSM-5), which is based on having at least two of the following eleven criteria: hazardous use, social or interpersonal problems related to use, neglected significant roles due to use, withdrawal, tolerance, use of more significant amounts, repeated attempts to quit or control use, much time spent using, physical or psychological problems related to use, activities given up using the drug, and craving. The severity of OUD was further classified into mild (2–3 criteria), moderate (4–5 criteria), and severe (6 or more criteria).

Psychological distress was measured by a standard K10 questionnaire. The K10 questionnaire includes 10 questions to screen for mental distress. It is a simple screening tool which is not used for making a diagnosis. Each response in the K10 questionnaire was rated on a 5-point Likert scale from 0 as “never” to 4 as “always”, with the total K10 scores ranging from 0 to 40. Based on the population and purpose of the study, different cutoffs have been proposed. We used a cutoff of 12 to define mild to severe psychological distress based on the K10 score validated for Iranian population, which has an area under the curve of 0.92 for detecting severe mental illness, with a sensitivity of 86% and a specificity of 83% [25–28].

### Statistical analysis

Opiate dose, frequency, type and route were analyzed at two time periods: “prior use” was reported on the baseline questionnaire administered in 2004–2008 (depending on the exact enrolment date), and “current use” was based on the new questionnaire administered in 2017. The doses were reported using a local using called “nokhod” which is almost equal to 0.2 grams [29]. For each time period (prior and current), we used the last reported daily use and number of days a person used during the week to calculate grams per week and categorized prior and current doses into quartiles. We also calculated dose change between the baseline interview and 2017, which were separated by 10–13 years. We. We also categorized the change of route of use into 4 groups: continued smoking, continued oral use, switch from smoking to oral use, and change from oral use to smoking. In 38 and 40 participants, dose and route change could not be calculated, respectively, due to missing data at either period. Age, sex, marital status, place of residence, education, BMI, and socioeconomic Status (SES) were extracted from the baseline questionnaire [30].

The descriptive statistics are presented as raw numbers, percentages, mean, and standard deviation or median and interquartile range whichever was appropriate. Univariate analysis of factors associated with OUD was done using Chi-square test for categorical variables and t-test for continuous variables. We used univariate ordered logistic regression to analyze the trends of these variables across categories of OUD severity (from mild to severe).

We investigated the independent role of opiate use patterns on OUD and OUD severity as the outcomes of interest, adjusted for potential confounders, using three different models: one for prior use, another for current use, and the third for the change between the two time periods. The models with OUD as an outcome of interest were examined through multivariate logistic regression (present/absent). In the models with OUD severity as an outcome we excluded those without an OUD diagnosis and used ordered logistic regression since the outcome had three levels (mild/moderate/severe). The odds ratio in ordered logistic regression reflects the odds of a one-step increase in the three levels of severity associated with the independent variables (opiate use pattern). All models were adjusted for age, age of starting opiate use, sex, place of residence (rural/urban), socioeconomic status (quartiles) and psychological distress K10 score. Stata statistical software, version 17 (StataCorp, College Station, TX) was used to perform analyses, and a p-value less than 0.05 was considered statistically significant.

## Results

Among 451 subjects (mean age 61.2 ± 6.6 years, 84.7% males), all used raw (teriak) or refined opium (shireh), and 261 (57.9%) were diagnosed as having opioid use disorder (OUD) in their lifetime based on DSM-5 criteria. Among opiate users with and without OUD, the most common DSM-5 criteria were withdrawal symptoms (94.3% and 48.4%, respectively), repeated attempts to quit or control use (73.5% and 49.5%, respectively), and craving (57.1% and 15.8%, respectively). OUD was mild in 144 (55.7%) of the individuals with OUD, moderate in 71 (27.2%) and severe in 46 (17.6%). As expected, the frequency of all criteria increased in people with more severe OUD. The details of DSM-5 criteria by OUD diagnosis and its severity are presented in Table 1.

**Table 1:** Prevalence of DSM-5 criteria among opiate users with and without opioid use disorder (OUD) and by OUD severity

DSM-5 criteria	Opiate users without OUD (N = 190)	Opioid use disorder			
		*All* *(N = 261)*	*Mild* *(N = 144)*	*Moderate* *(N = 71)*	*Severe* *(N = 46)*
Hazardous use, n (%)	49 (25.7)	117 (44.8)	44 (30.5)	39 (54.9)	34 (73.9)
Social/interpersonal problems related to use, n (%)	6 (3.1)	30 (11.4)	1 (0.6)	5 (7.0)	24 (52.1)
Neglected major roles to use, n (%)	6 (3.1)	38 (14.5)	3 (2.0)	10 (14.0)	25 (54.3)
Withdrawal, n (%)	92 (48.4)	246 (94.2)	133 (92.3)	69 (97.1)	44 (95.6)
Tolerance, n (%)	18 (9.4)	52 (19.9)	8 (5.5)	19 (26.7)	25 (54.3)
Used larger amounts/longer, n (%)	11 (5.8)	56 (21.4)	8 (5.5)	22 (30.9)	26 (56.5)
Repeated attempts to quit/control use, n (%)	94 (49.4)	191 (73.4)	94 (65.2)	58 (81.6)	39 (86.6)
Much time spent using, n (%)	14 (7.3)	63 (24.1)	9 (6.2)	21 (29.5)	33 (71.7)
Physical/psychological problems related to use, n (%)	6 (3.1)	34 (13.0)	5 (3.5)	12 (16.9)	17 (36.9)
Activities given up to use, n (%)	3 (1.5)	23 (8.9)	2 (1.4)	5 (7.2)	16 (34.7)
Craving, n (%)	30 (15.7)	149 (57.0)	55 (38.1)	52 (73.2)	42 (91.3)

OUD: opioid use disorder

Individuals with OUD were significantly younger (60.6 ± SD vs. 62.1 ± SD years old) than those without OUD, were more likely to live in rural area, had lower socioeconomic status, lower BMI, and higher psychological distress (Table 2). There were no statistically significant differences in sex, marital status, education, cigarette use, and history of ever alcohol use between the two groups. However, cigarette smoking, in addition to younger age and higher education and psychological distress were significantly associated with OUD severity (Supplementary Table 1). The K10 scale for psychological distress was positive in 31.7% of the study participants. Individuals with OUD had higher scores of psychological distress, and were more likely to have a score of equal to or above the cut-off of 12 (*p* < 0.01). In subjects with OUD, the odds of positive K10 scores were more than two times the odds in those without it (OR = 2.25; 95%CI: 1.44–3.52, *p* < 0.001). Similar, but stronger associations were found among individuals with severe OUD. These associations changed slightly after adjustment for current and prior opiate dose and route and their changes between these periods (Supplementary Table 2).

**Table 2:** Demographic characteristics of opiate users with and without opioid use disorder

Demographic Parameters		Total (N = 451)	Opioid use disorder	
			*Negative* *(N = 190)*	*Positive* *(N = 261)*
Age (Mean ± SD)		61.1 ± 6.5	62.0 ± 6.6	60.5 ± 6.4*
Sex, n (%)	Male	382 (84.7)	162 (85.3)	220 (84.3)
	Female	69 (15.3)	28 (14.7)	41 (15.7)
Marital status, n (%)	Single	20 (4.4)	7 (3.7)	13 (5.0)
	Married	431 (95.6)	183 (96.3)	248 (95.0)
Education, n (%)	Illiterate	167 (37.0)	67 (35.3)	100 (38.3)
	< 9y	195 (43.2)	88 (46.3)	107 (41.0)
	>= 9y	89 (19.8)	35 (18.4)	54 (20.7)
Cigarette user, n (%)	No	294 (65.1)	129 (67.9)	165 (63.2)
	Yes	157 (34.9)	61 (32.1)	96 (36.8)
History of ever alcohol use, n (%)	No	389 (86.2)	164 (86.3)	225 (86.2)
	Yes	62 (13.8)	26 (13.7)	36 (13.8)
Residence, n (%)	Urban	211 (46.8)	104 (54.7)	107 (41.0)**
	Rural	240 (53.2)	86 (45.3)	154 (59.0)
Socioeconomic Status, n (%)	Q1	77 (17.1)	23 (12.1)	54 (20.7)*
	Q2	88 (19.5)	38 (20.0)	50 (19.2)
	Q3	128 (28.4)	57 (30.0)	71 (27.2)
	Q4	158 (35.0)	72 (37.9)	86 (32.9)
BMI (Mean ± SD)		24.6 ± 4.7	25.3 ± 4.5	24.1 ± 4.7**
K10 score Median (IQR)		5 (2–11)	4 (1–9)	6 (2–11)**
Psychological distress, n (%)	K10 < 12	308 (68.3)	147 (77.3)	161 (61.7)**
	K10 > = 12	143 (31.7)	43 (22.7)	100 (38.3)

SD: standard deviation, BMI: body mass index, K10 score: Kessler 10 score

* *P* < 0.05, ** *P* < 0.01

Table 3 compares the patterns of opiate use between those with and without OUD and by OUD severity. Individuals with OUD started opiate use at an earlier age (35.1 ± 12.4 vs. 38.8 ± 12.6 years). As OUD severity increased, the start age decreased to 30.2 (SD = 10.0) in severe cases. Prior and current opiate doses were both significantly associated with the presence and severity of OUD (Table 3). Among individuals whose dose change during the follow-up could be calculated, 228 (55.2%) increased their dose, 66 (16.0%) and 107 (25.9%) reported unchanged and decreasing opiate doses, respectively, and 12 (2.9%) quit using opiates. As a result, average weekly dose increased from 3.6 ± 3.9 g at baseline to 4.6 ± 4.3 g. Individuals with OUD were more likely to have increased their opiate dose compared to those without OUD (59.8% vs. 48.8%, respectively) and reported a significantly higher average increase in dose (mean increase: 1.7 vs. 0.13 g per week, *p* < 0.01). Individuals with OUD were more likely to consume opium orally, both currently and more than 10 years prior, however the change in route was not associated with OUD severity. There were no significant differences between the types of current and prior opiates used by those with and without OUD.

**Table 3:** Opiate use characteristics by presence and severity of opioid use disorder

Opiate use characteristics		Opiate users w/o Disorder (N = 190)	Opioid use disorder				P for trend by severity†
			*All* *(N = 261)*	*Mild* *(N = 144)*	*Moderate* *(N = 71)*	*Severe* *(N = 46)*	
Age of start opiate use (Mean ± SD)		38.8 ± 12.6	35.1 ± 12.4**	37.9 ± 13.0	32.7 ± 11.0	30.1 ± 9.9	< 0.001
Current opiate dose, n (%) (gram per week) ^a^	Q1 (< 1.4)	92 (49.4)	63 (24.7)**	38 (26.7)	12 (17.1)	13 (30.2)	0.049
	Q2 (1.4–2.8)	34 (18.2)	52 (20.3)	33 (23.2)	16 (22.8)	3 (6.9)	
	Q3 (2.8-7.0)	39 (20.9)	71 (27.8)	40 (28.1)	23 (32.8)	8 (18.6)	
	Q4 (> 7.0)	21 (11.2)	69 (27.0)	31 (21.8)	19 (27.1)	19 (44.1)	
Prior opiate dose, n (%) (gram per week) ^b^	Q1 (< 0.6)	6 (35.5)	55 (21.4)**	37 (26.2)	12 (17.1)	6 (13.3)	< 0.001
	Q2 (0.6–2.8)	56 (30.6)	85 (33.2)	54 (38.3)	21 (30.0)	10 (22.2)	
	Q3 (2.8–10.5)	32 (17.4)	46 (17.9)	22 (15.6)	13 (18.5)	11 (24.4)	
	Q4 (> 10.5)	30 (16.3)	70 (27.3)	28 (19.8)	24 (34.2)	18 (40.0)	
Change in dose (gram/week), mean (SD) ^c^		0.13 (4.6)	1.7 (4.9)**	2.1 (4.9)	1.2 (4.7)	1.5 (5.2)	0.15
Current pattern of opiate use, n (%)	Non- daily	38 (20.0)	26 (10.0)**	14 (9.8)	3 (4.3)	9 (19.5)	0.334
	Daily	152 (80.0)	235 (90.0)	130 (90.2)	68 (95.7)	37 (80.5)	
Prior pattern of opiate use, n (%) ^b^	Non daily	74 (40.4)	80 (31.2)*	49 (34.7)	21 (30.0)	10 (22.2)	0.128
	Daily	109 (59.5)	176 (68.7)	92 (65.2)	49 (70.0)	35 (77.7)	
Current type of the opiate, n (%) ^d^	Opium only	88 (50.2)	109 (46.1)	67 (50.3)	31 (46.2)	11 (30.5)	0.070
	Shire ± opium	87 (49.7)	127 (53.1)	66 (49.6)	36 (53.7)	25 (69.4)	
Prior type of the opiate, n (%) ^b^	Opium only	162 (88.5)	219 (85.5)	124 (87.9)	59 (84.2)	36 (80.0)	0.180
	Shire ± opium	21 (11.4)	37 (14.4)	17 (12.0)	11 (15.7)	9 (20.0)	
Current route of opiate use, n (%) ^e^	Smoke only	99 (55.3)	82 (33.8)**	51 (37.7)	24 (35.2)	7 (17.9)	< 0.01
	Oral only	74 (41.3)	132 (54.5)	73 (54.0)	37 (54.4)	22 (56.4)	
	Dual	6 (3.3)	28 (11.5)	11 (8.1)	7 (10.2)	10 (25.6)	
Prior route of opiate use, n (%) ^b^	Smoke only	146 (79.7)	174 (67.9)*	100 (70.9)	47 (67.1)	27 (60.0)	0.165
	Oral only	34 (18.5)	74 (28.9)	37 (26.2)	22 (31.4)	15 (33.3)	
	Dual	3 (1.6)	8 (3.1)	4 (2.8)	1 (1.4)	3 (6.6)	
Change in route, n (%) ^d^	Still smoke	87 (50.2)	79 (33.1)**	49 (36.8)	24 (35.8)	6 (15.7)	0.075
	Still oral	30 (17.3)	72 (30.2)	37 (27.8)	22 (32.8)	13 (34.2)	
	Change from smoke	50 (28.9)	78 (32.7)	43 (32.3)	20 (29.8)	15 (39.4)	
	Change from oral	6 (3.4)	9 (3.7)	4 (3.0)	1 (1.4)	4 (10.5)	

SD: standard deviation, BMI: body mass index.

Number of dual users of opium and shireh is 13 in current status and 9 in baseline status.

The missing values are as follows: a = 10, b = 12, c = 38, d = 40, e = 30

* *P* < 0.05, ** *P* < 0.01

† Using univariate ordered logistic regression across categories of OUD severity (from mild to severe)

Table 4 presents the results of multivariate models assessing the associations between current and prior opiate use patterns and OUD presence and severity, adjusted for age, opiate start age, sex, place of residence, socioeconomic status, and K10 score. Prior opiate dose was independently associated with the presence of OUD and OUD severity (p for trend < 0.05), however current opiate dose was associated with OUD presence and not its severity. Each ten grams per week increase in opiate dose during the study period almost tripled the odds of OUD presence (OR = 3.18; 95%CI: 1.79–5.63) without a significant effect on OUD severity. The odds of OUD and severe OUD increased up to three times in people who currently used opiates by both oral and smoking routes, i.e. dual use (OR = 2.96; 95%CI: 1.11–7.92, and OR = 2.65; 95%CI: 1.07–6.54, respectively). Prior route of use did not significantly predict OUD and its severity. Individuals who previously used oral opiates and still had the same habit at the time of follow-up had the highest odds of OUD (OR = 2.97; 95%CI:1.55–5.71) compared to subjects who continued smoking opiates. The change from smoking to oral route had the second highest odds ratios for OUD (OR = 1.87; 95%CI: 1.10–3.17). The change in route was not associated with OUD severity.

**Table 4:** Multivariable regression models predicting presence and severity of opioid use disorder (OUD) as outcomes

Parameters		Opioid use disorder as the outcome^†^ OR (95% CI)	Severity of OUD as the outcome ^†^ OR (95% CI)
Current opiate dose	Q1 (< 1.4)	Ref	Ref
	Q2 (1.4–2.8)	2.65 (1.41–4.97)**	0.81 (0.33-2.00)
	Q3 (2.8-7.0)	3.55 (1.98–6.37)**	1.19 (0.52–2.70)
	Q4 (> 7.0)	5.87 (2.96–11.65)**	1.88 (0.82–4.33)
*P trend*		< 0.01	0.054
Prior opiate dose	Q1 (< 0.6)	Ref	Ref
	Q2 (0.6–2.8)	1.81 (1.04–3.16)*	1.37 (0.64–2.93)
	Q3 (2.8–10.5)	1.38 (0.72–2.65)	2.19 (0.91–5.23)
	Q4 (> 10.5)	2.21 (1.20–4.09)*	2.51 (1.15–5.47)*
*P trend*		< 0.05	< 0.05
Change in dose (for each 10-gram per week change)		3.18 (1.79–5.63)**	1.01 (0.58–1.76)
Current opiate route	Smoke only	Ref	Ref
	Oral only	1.54 (0.93–2.54)	1.29 (0.66–2.51)
	Dual	2.96 (1.11–7.92)*	2.65 (1.07–6.54)*
*P trend*		< 0.01	0.054
Prior opiate route	Smoke only	Ref	Ref
	Oral only	1.35 (0.76–2.40)	1.17 (0.60–2.25)
	Dual	1.28 (0.29–5.53)	1.21 (0.27–5.25)
*P trend*		0.295	0.593
Change in route	Still smoke	Ref	Ref
	Still oral	2.97 (1.55–5.71)**	1.88 (0.86–4.10)
	Change from smoke	1.87 (1.10–3.17)*	1.44 (0.72–2.85)
	Change from oral	1.56 (0.48–5.04)	4.09 (0.95–17.52)

^†^ All models adjust for age, age of start opiate use, sex, residence, socioeconomic status, and K10 score, dose, and route.

CI, confidence interval; OR, odd ratio.

* *P* < 0.05, ** *P* < 0.01

## Discussion

More than half (57.9%) of the people who had used opiates for a long time in our study were diagnosed with an opiate use disorder. Opiate users with an OUD diagnosis were relatively younger than those without OUD and had started using at an earlier age. Other factors associated with OUD included living in rural areas, having low socioeconomic status and having a lower BMI. Younger and more educated opiate users, and those who smoked cigarettes, were more likely to have severe OUD. Individuals with OUD had higher psychological distress scores compared with opiate users without such diagnosis. Patterns of opiate use were also different between individuals with and without OUD: opiate users with OUD used higher doses and were more likely to increase their dose during the follow-up, and OUD diagnosis was more common in those using opiates orally instead of smoking.

Our study confirms that the widespread consumption of opiates has led to OUD and is a major health problem in this population. In the U.S., the prevalence of OUD has been estimated at around 0.7% in people aged 25 years and above [31], and in the Netherlands, the prevalence of opioid use disorder other than heroin was reported 0.0056% [32, 33]. The results of the 2011 Iranian Mental Health Survey which showed a prevalence of 3.02% for opiate use in the past year, and 2.23% for OUD [18]. We believe that the overall rate of OUD in our study population is higher than these estimates, as we studied lifetime prevalence which is naturally higher compared with the past year definition used in the survey. Also the prevalence of opiate use in Iran follows a geographic pattern and the provinces in the south and northeast (where our study was conducted) have higher rates compared to other regions of the country. Finally, we investigated people between 40 and 75 years, while the 2011 national study was conducted on 15 to 64 years group, and lifetime prevalence of OUD increases overtime.

In Iran, opium is sometimes considered a “soft drug” due to its widespread use, but the fact that almost three out of five long-term opium users were clinically diagnosed as having an OUD is in contrast with this notion. OUD is associated with poor mental health and a number of important psychological comorbidities. Opiate use increases the risk of mood disorders, suicide attempts, and violent behavior [34–36]. Other substance uses and severe mental disorders are commonly associated with OUD [37]. Mood and anxiety disorders may precede the substance use and may even be the cause of abuse, making the diagnosis and treatment even more complex [34, 38]. People with psychological comorbidities are more prone to seek a substance to resolve their undesirable psychological symptoms [39]. Our study did not include an assessment of these mental comorbidities, but we screened for psychological distress using a validated questionnaire. K10 were first developed to screen for more recent non-specific symptoms of serious mental disorders, i.e. anxiety disorders, mood disorders, and psychotic disorders [25, 40]. People with higher psychological distress are more likely to seek outpatient mental health care, be under psychological treatment in the future, and have higher mortality [26, 27]. Nearly 32% of opiate users in this study had psychological distress, and individuals with OUD were twice more likely to have psychological distress after adjusting for opiate use patterns. In a study from Nepal, half of all kinds of substance users in drug rehabilitation centers had psychological distress [41]. Similar findings have also been reported for cannabis and nonmedical use of prescription opioids [42, 43]. Our cross-sectional analysis would not allow us to conclude a causal relationship, since it is not clear if the psychological distress preceded the OUD diagnosis or occurred because of it. However, the high prevalence of this comorbidity underlines the importance of appropriate psychosocial support for opiate users, especially those with OUD.

Opium consumption is a known carcinogen [9], and recent studies have found that it can increase the risk of premature mortality from different causes [12, 44–46]. These risks increase with the dose and duration of use, putting users with OUD at an even greater risk. Opium users have also been shown to be exposed to very high levels of lead, probably as a result of opium adulterations to increase weight. Lead exposure among users is dose-dependent and is seen mainly among those who used opium orally [47]. Lead is a probable carcinogen and a risk factor for cardiovascular diseases, and users with OUD are more likely to have high blood lead levels due to their longer and higher use. Moreover, in our study people with OUD were more likely to take opium orally in both periods and keep their habit of eating opiates. Oral consumption is easier than inhalation, and enables the heavy user to consume opiates regardless of time and location. These hazards are aggravated by the fact that the most common OUD symptoms in our population of opiate users were withdrawal symptoms and repeated (failed) attempts to quit or control use, meaning that a large proportion of these long-term users will continue using for a long time, maybe for the rest of their lives.

Certain groups of opiate users were at increased risk of developing OUD in our study. Opiate users with OUD were on average younger than those without it, because they may live shorter, and had started using at an earlier age. In the U.S., National Epidemiologic Study on Alcohol and Related Conditions reported similar finding and suggested implementing programs to prevent the initiation of opioid use with an emphasis on younger age groups [48]. A comparative study on three cohorts of Millennials (1979-96), Generation X (1964-79), and Baby Boomers (1949-64) reported higher odds of nonmedical prescription opioid use and use disorder in Millennials [49, 50]. Younger individuals become dependent to a substance faster than the older age groups [51]. Besides, coping with social and psychological challenges is one of the proposed reasons for the higher frequency of OUD among younger age groups [52]. Prioritizing younger and long-time users for opiate use treatment consultations can slow the upward trend of opiate use disorder in the future.

We showed that rural users and those in lower SES levels were more likely to have OUD than urban users with higher SES scores. Our finding on socioeconomic status was in line with the Iranian household Mental Health survey, in which subjects with higher SES were at lower risk of opioid dependence than those at lower levels [17]. Also, the U.S. National Epidemiologic Study on Alcohol and Related Conditions reported incomes at or above 40,000 U.S. dollars as a protective factor for OUD [48]. Opium is inexpensive, which makes it affordable for people with low income even in rural areas.

### Limitations

We studied a sizeable number of opiate users and evaluated clinical OUD by DSM-5 criteria in a non-clinical setting using a validated questionnaire. The availability of detailed data on the lifetime pattern of opiate use and other lifestyle variables in the context of a prospective cohort study is another strength of this study. Comparing these data in the baseline and after more than 10 years of follow-up enabled us to investigate the impact of changes in opiate use on OUD. However, the OUD questionnaire was only available at the follow-up visit, and we had not assessed OUD at the baseline, which is the main limitation of our study. Assessment of psychological distress was also done at the follow-up. Another limitation of this study was insufficient information on other comorbidities such as depression and anxiety for further adjustment. However, we used Kessler score for evaluating psychologic distress and found it quite useful in a study of a high-risk group. Also, we were not able to investigate some previously proposed OUD risk factors like personal and psychological traits due to lack of data.

## Conclusions

In conclusion, we found that more than half of the subjects with a history of long-term opiate use had OUD, which means that it is probably one of the most common mental disorders in this population. In addition to the known long-term health hazards, we showed that OUD was associated with psychological distress. Identifying groups at high risk of OUD, such as those who start using at an earlier age, live in underprivileged conditions, and use higher doses and increase their dose is important in planning appropriate prevention strategies to lower the physical and mental burden of OUD.

### Electronic supplementary material

Below is the link to the electronic supplementary material.

Supplementary Material 1

## Data Availability

The datasets used and/or analyzed during the current study are available from the corresponding author on reasonable request.
