# Supplementary material for: Prevalence and determinants of opioid use disorder among long-term opiate users in Golestan Cohort Study
Source: BMC Psychiatry. 2023 Dec 21;23:958. doi: 10.1186/s12888-023-05436-x (PMC10734090; doi:10.1186/s12888-023-05436-x)
Supplement: Supplementary file 1 — Supplementary Material 1 [file 12888_2023_5436_MOESM1_ESM.docx]

| **Supplementary Table 1.**  Demographic characteristics of opiate users with and without opioid use disorder (OUD) and by OUD severity | | | | | | |
| --- | --- | --- | --- | --- | --- | --- |
| Demographic Parameters | | Opiate use w/o  Disorder  (N=190) | Opioid use disorder | | | P for trend by severity† |
|  |  |  | *Mild*  *(N=144)* | *Moderate*  *(N=71)* | *Severe*  *(N=46)* |  |
| Age (Mean± SD) | | 62.0 ± 6.6 | 61.7 ± 6.6 | 59.7 ± 6.4 | 58.2 ± 5.2 | <0.01 |
| Sex, n (%) | Male | 162 (85.3) | 121 (84.0) | 57 (80.3) | 42 (91.3) | 0.574 |
|  | Female | 28 (14.7) | 23 (16.0) | 14 (19.7) | 4 (8.7) |  |
| Marital status, n (%) | Single | 7 (3.7) | 6 (4.2) | 6 (8.5) | 1 (2.2) | 0.842 |
|  | Married | 183 (96.3) | 138 (95.8) | 65 (91.5) | 45 (97.8) |  |
| Residence, n (%) | Urban | 104 (54.8) | 64 (44.4) | 22 (31.0) | 21 (45.7) | 0.444 |
|  | Rural | 86 (45.2) | 80 (55.6) | 49 (69.0) | 25 (54.3) |  |
| Education, n (%) | Illiterate | 67 (35.3) | 61 (42.4) | 26 (36.6) | 13 (28.3) | <0.01 |
|  | <9y | 88 (46.3) | 63 (43.7) | 30 (42.3) | 14 (30.4) |  |
|  | >= 9y | 35 (18.4) | 20 (13.9) | 15 (21.1) | 19 (41.3) |  |
| BMI, n (%) | Underweight | 13 (6.8) | 15 (10.4) | 7 (9.9) | 5 (10.9) | 0.911 |
|  | Normal | 78 (41.1) | 72 (50.0) | 30 (42.3) | 26 (56.5) |  |
|  | Overweight | 70 (36.9) | 43 (29.9) | 21 (29.5) | 14 (30.4) |  |
|  | Obese | 29 (15.2) | 14 (9.7) | 13 (18.3) | 1 (2.2) |  |
| Socioeconomic Status, n (%) | Q1 | 23 (12.1) | 31 (21.5) | 15 (21.1) | 8 (17.4) | 0.453 |
|  | Q2 | 38 (20.0) | 32 (22.2) | 8 (11.3) | 10 (21.8) |  |
|  | Q3 | 57 (30.0) | 35 (24.3) | 22 (31.0) | 14 (30.4) |  |
|  | Q4 | 72 (37.9) | 46 (32.0) | 26 (36.6) | 14 (30.4) |  |
| K10 score Median (IQR) |  | 4 (8) | 6 (10.7) | 6 (9) | 10.5 (12) | 0.224 |
| Psychological distress, n (%) | K10 <12 | 147 (77.4) | 95 (66.0) | 45 (63.4) | 21 (45.7) | <0.05 |
|  | K10 >=12 | 43 (22.6) | 49 (34.0) | 26 (36.6) | 25 (54.3) |  |
|  |  |  |  |  |  |  |
| History of ever alcohol use, n (%) | No | 164 (86.3) | 127 (88.2) | 59 (83.1) | 39 (84.8) | 0.567 |
|  | Yes | 26 (13.7) | 17 (11.8) | 12 (16.9) | 7 (15.2) |  |
| Cigarette user, n (%) | No | 129 (67.9) | 103 (71.5) | 44 (62.0) | 18 (39.1) | <0.001 |
|  | Yes | 61 (32.1) | 41 (28.5) | 27 (38.0) | 28 (60.9) |  |
| SD: standard deviation, BMI: body mass index, K10: Kessler 10  * P<0.05, ** P <0.01  † Using univariate ordered logistic regression across categories of OUD severity (from mild to severe) | | | | | | |

| **Supplementary Table 2.**  Multivariable regression models predicting psychological distress based on the K10 score | | | | | |
| --- | --- | --- | --- | --- | --- |
|  |  | Model 1 | Model 2 | Model 3 | Model 4 |
| OUD |  | 2.25 (1.44-3.52)** | 2.17 (1.32-3.56)** | 2.18 (1.37-3.45)** | 2.11 (1.29-3.45)** |
| OUD severity | Mild OUD | Ref | Ref | Ref | Ref |
|  | Moderate OUD | 1.14 (0.60-2.15) | 1.25 (0.63-2.44) | 1.21 (0.62-2.35) | 1.16 (0.59-2.27) |
|  | Severe OUD | 2.62 (1.28-5.37)** | 3.62 (1.53-8.57)** | 2.95 (1.38-6.32)** | 3.10 (1.33-7.25)** |
| *P trend* | | <0.05 | <0.01 | <0.01 | <0.05 |
| OUD: opioid use disorder  Model 1: Adjusted for age, age of start opiate use, sex, residence, socioeconomic status  Model 2: Model 1 + current opiate dose, current opiate route  Model 3: Model 1 + baseline opiate dose, baseline opiate route  Model 4: Model 1 + change in dose, change in route | | | | | |
